# Access to Care and Services Among U.S. Rural Veterans With and Without Disabilities: A National Study

**DOI:** 10.3390/healthcare13030275

**Published:** 2025-01-30

**Authors:** Emre Umucu, Teresa Ann Granger, Bryan Weichelt, Traci McGee, Gloria Lee, Aylin Celik Zencir, Jim Yates, John Barnas, Crystal Barter, Beatrice Lee

**Affiliations:** 1 Department of Public Health Sciences, The University of Texas at El Paso, El Paso, TX 79968, USA; 2 South Texas VA Medical Center, San Antonio, TX 78208, USA; 3 Department of Educational Studies in Psychology, Research Methodology, and Counseling, University of Alabama, Tuscaloosa, AL 35487, USA; 4 Tuscaloosa VA Medical Center, Research & Development, Tuscaloosa, AL 35401, USA; 5 Marshfield Clinic, Marshfield, WI 54449, USA; 6 Department of Counseling, Educational Psychology, and Special Education, Michigan State University, East Lansing, MI 48824, USA; 7 Department of Rehabilitation Psychology and Special Education, UW-Madison, Madison, WI 53706, USA; 8 Michigan Center for Rural Health, East Lansing, MI 48824, USA; 9 Department of Rehabilitation Sciences, The University of Texas at El Paso, El Paso, TX 79968, USA

**Keywords:** rural veterans, healthcare access, service-connected disability, chronic conditions, health disparities

## Abstract

**Background:** Access to healthcare is a significant challenge for rural veterans, especially those with chronic conditions; yet, research on their specific barriers remains limited. This study had three objectives: (1) to develop and validate the *Rural Access to Care and Services Scale* (RACSS) as a simple tool to assess access to physical, mental, and social care and services; (2) to examine whether minority veterans face greater barriers to care; and (3) to test whether RACSS scores are associated with psychosocial outcomes as theoretically expected. **Methods:** Data were collected from 500 rural veterans in the U.S. through a survey assessing demographic information, service-connected disability status, clinical conditions, and access to healthcare. First, the RACSS was developed and validated using Exploratory Factor Analysis (EFA) and Confirmatory Factor Analysis (CFA). Second, to assess the reliability of the RACSS, internal consistency was evaluated using Cronbach’s alpha. Finally, independent sample *t*-tests were conducted to explore the relationships between access to healthcare and services and participants’ minority status. **Results:** The scale demonstrated a one-factor structure with excellent model fit indices and high internal consistency (α = 0.89). Veterans from minoritized racial and ethnic groups reported significantly higher difficulties in accessing healthcare and services. **Conclusions:** The RACCS provides a reliable tool to measure access to healthcare and services among rural veterans. The findings highlight the critical need for targeted interventions to improve access to healthcare and services, especially for veterans from minoritized racial and ethnic groups. Enhancing healthcare and service delivery in rural areas is essential to reducing disparities and improving health outcomes for this underserved population.

## 1. Introduction

Access to healthcare and services is a critical determinant of health outcomes [1]. Previous studies have underscored the importance of accessing healthcare in rural areas, particularly for veterans [2,3,4]. Rural veterans represent a unique and vulnerable group due to their service-related chronic health conditions. For instance, Weeks et al. [5] reported that the prevalence of physical and mental health disabilities is higher among rural veterans than their urban counterparts. Supporting this, higher rates of service-related disability have been observed among rural veterans, particularly in the Midwest and Northeast regions, compared to urban veterans [6]. The Veteran Health Administration (VHA) healthcare system is a valuable resource for veterans regarding access to healthcare, distinct from other healthcare systems due to its funding by the U.S. federal government [7]. However, residing in rural areas may impede access to VHA healthcare. For example, rural veterans make up 24% (4.4 million) of the U.S. veteran population, with 48% (2.11 million) enrolled in VHA healthcare [8]. Whilst this enrollment rate is higher than that of urban veterans, at 41%, it still comprises only roughly half of the rural veteran population. The VHA has made significant efforts to expand access to care for rural veterans through initiatives such as community care programs, where the VA provides care to veterans through other non-VA providers [8], telehealth services, and mobile clinics [9,10,11].

Despite these efforts having improved access to some extent, significant gaps remain, particularly for those who face multiple barriers to care and services. For instance, rural veterans face a multitude of challenges in accessing healthcare, including geographic isolation, limited availability of health professionals, and transportation difficulties [2,3,4,9]. These challenges are further compounded for veterans who often require specialized care for conditions such as post-traumatic stress disorder (PTSD), traumatic brain injuries, and other service-related health issues [12]. Additionally, rural veterans may also experience social and economic barriers, including lower incomes, limited social support, and higher rates of mental health conditions, which can further exacerbate their difficulties in accessing care [3,4,13,14]. These disparities are concerning, since not having access to healthcare may result in many adverse outcomes. For instance, Shiner et al. [15] have reported that death by suicide among rural veterans is higher than among their urban counterparts. Similarly, rural veterans experience lower health-related quality of life scores in physical health dimensions compared to urban veterans [16]. The intersection of rural residence with minority status or having a service-connected disability may lead to even greater disparities in healthcare access. Rural veterans from minoritized racial and ethnic groups may face discrimination or cultural barriers that hinder their access to care [17], while those with service-connected disabilities may encounter additional challenges related to the complexity of their healthcare needs [5,18]. Considering these findings, understanding their unique needs and the barriers they face is essential.

Admittedly, rural veterans and the general rural population may share some challenges in accessing healthcare; however, service-related disabilities often result in unique healthcare needs that are not typically experienced by the broader rural population (e.g., exposure to burn pits [19]). Also, previously mentioned disparate barriers highlight the need for a comprehensive understanding of healthcare access in this population, as well as targeted interventions that address the specific needs of these subgroups. The development of a reliable and valid tool to measure access to healthcare and services among rural veterans is therefore essential. Such a tool can provide valuable insights into the specific barriers faced by this population, facilitate the monitoring of access to care, and inform the design of interventions aimed at improving healthcare and service delivery. The *Rural Access to Care and Services Scale* (RACSS) developed in this study aims to fill this gap by providing a comprehensive measure of healthcare access that accounts for the unique challenges faced by rural veterans.

Consequently, the present study has two primary objectives. First, it seeks to develop and validate the RACSS among a sample of rural veterans, as a brief tool to measure access to care and services. This scale is intended to capture the nature of access to healthcare and services, including physical, mental, and social dimensions. Second, the study also aims to examine whether minoritized veterans have lower levels of access to healthcare and services. Finally, we tested whether the RACSS scores were associated with psychosocial outcomes in the expected theoretical direction. By addressing these objectives, this study contributes to the growing body of literature on rural access to healthcare and services and offers practical tools for assessing and improving healthcare services for rural veterans. The findings are expected to provide critical insights into the barriers faced by this population and inform the development of targeted interventions and policies that will aim at reducing healthcare disparities and improving health outcomes for rural veterans.

## 2. Methods

### 2.1. Procedure

We recruited veterans living in rural areas after receiving approval from the Institutional Review Board. Eligible participants had the following characteristics: (a) a veteran 18 years old or older and (b) living in a rural setting in the U.S. We utilized convenient sampling by using social media materials and personal connections to collect data. In order to improve data quality, we utilized attention check items (e.g., “Select correct responses: five plus two = seven.”; “Select the color option below: Car.”). We removed participants who failed the attention check. We had a total of 1022 participants who initiated our survey. A total of 522 participants were removed from the dataset due to failing attention check items and/or not completing the survey, resulting in a total of 500 completed responses.

#### Participants

Our sample consisted of a total of 500 veterans living in rural settings. Participants’ mean age was 34.86 (SD = 10.99). Most participants were male (*N* = 401; 80.2%) and non-Hispanic White (*N* = 357; 71.4%), followed by Black (*N* = 80; 16%), American Indian or Alaska Native (*N* = 29; 5.8%), Native Hawaiian or Pacific Islander (*N* = 14; 2.8%), Asian (*N* = 13; 2.6%), and others (*N* = 7; 1.4%). About 20% of participants identified as Latinx. Regarding educational attainment and employment, most participants had at least a high school degree (96.4%) and were employed (73.6%).

### 2.2. Materials

We used a demographic questionnaire to gather data on participants’ age, gender, race, ethnicity (i.e., “Are you of Hispanic, Latino, or Spanish origin?”), and education. Regarding information on access to healthcare and services, there are two sections.

The rural access to care and services was measured by developing an eight-item scale with a stem of “Due to living in a rural area”, followed by the specific type of services (e.g., “I have not been able to access physical health care services”). These items were developed based on a review of the literature on barriers and challenges faced by rural veterans. The scale utilized a Likert scale, with response options ranging from 1 (strongly disagree) to 5 (strongly agree), with higher scores indicating lower levels of access to care and services. (Please refer to the Supplementary Materials document for instructions on how to use the scale).

Service-connected disability was measured by asking participants the following question: “Do you have a service-connected disability rating?”. In order to measure whether participants had any physical or mental health conditions, each participant was also asked if they had any physical or mental health conditions (i.e., “Do you have any of the following conditions? [Check all that apply]”). These conditions were also listed based on a review of the literature on the common conditions experienced by veterans. We categorized these conditions as physical and mental health conditions. To examine whether our scale score correlated with willingness to seek psychological help, we used a single item: “I am willing to see a therapist for psychological help”.

### 2.3. Data Analysis

Our study utilized a random split-half technique to conduct an exploratory factor analysis (*N* = 247), as well as a confirmatory factor analysis (*N* = 253). An EFA and a CFA were conducted with SPSS 28. Reliability in terms of internal consistency was determined by calculating the reliability coefficient (Cronbach’s alpha) of the RACSS. Furthermore, an independent sample *t*-test was conducted to compare the access to healthcare and services of rural veterans and minority status. Finally, we conducted a correlation analysis to examine if the RACSS scores were correlated with willingness to seek psychological help, number of mental health conditions, and number of physical health conditions.

## 3. Results

### 3.1. Descriptive Statistics

Among 500 veterans living in rural areas, about 46% reported a service-connected disability rating. Anxiety (41%) was the most reported mental health condition, followed by depression (36%), PTSD (21%), bipolar disorder (15.4%), substance use disorders (6.4%), personality disorder (5.6%), and schizophrenia (4%). Migraine (20.6%) was the most reported physical health condition followed by tinnitus (16.2%), paralysis (14.6%), hearing loss (14%), musculoskeletal disease (11.2%), Alzheimer’s disease (2%), and others (3.8%). The mean number of physical health conditions and mental health conditions were 0.82 (SD = 1.06) and 1.29 (SD = 1.25), respectively.

### 3.2. Exploratory Factor Analysis

The 8 × 8 correlational matrix of the RACSS was subjected to a factor analysis. Our analysis results revealed a 0.90 (>0.60) KMO measure of sampling adequacy, with a significant Bartlett’s test of sphericity (*χ*^2^ (28, *N* = 247) = 887.36, *p* < 0.001), demonstrating that the variables are interrelated and appropriate to explore underlying factors. Both Cattell’s Scree test and Kaiser-Guttman’s criterion revealed a single factor, with factor loadings ranging from 0.69 to 0.78, as seen in Table 1. According to Hair et al. [20], factor loading above 0.50 is practically significant.

### 3.3. Confirmatory Factory Analysis

With the second sample (*N* = 253), in order to cross-validate the unidimensional nature of our newly developed scale, we utilized a CFA [21]. We used the following fit indices and criteria to test whether our model fit the data well: chi-square goodness-of-fit test (*χ*^2^), *χ*^2^/*df* (in the range of 1–3), the Comparative Fit Index (CFI; >0.95), the Tucker–Lewis Index (TLI; >0.95), the Standardized Root Mean Square Residual (SRMR; <0.05), and the Root Mean Square Error of Approximation (RMSEA; >0.08) [22,23].

**Figure 1:**
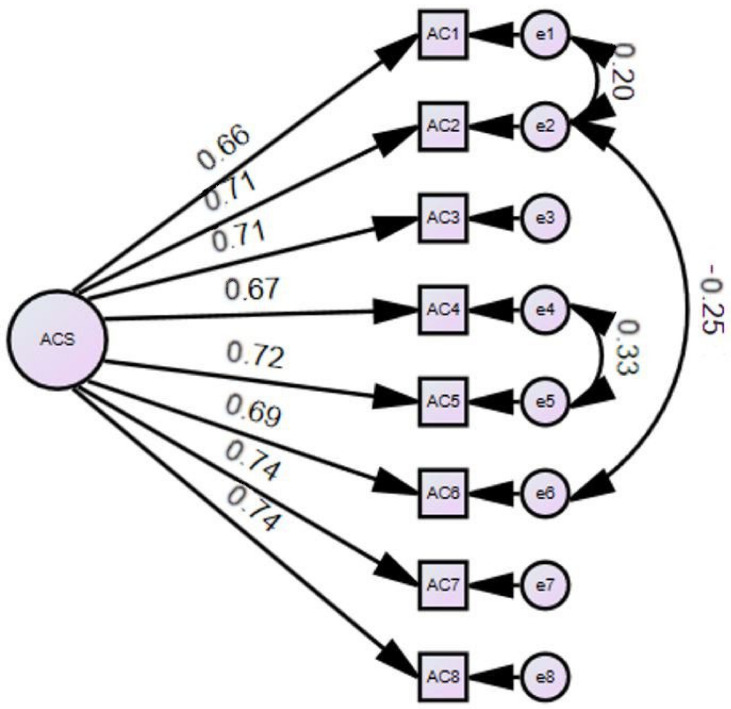
CFA results of the RACSS.

The initial one-factor CFA model indicated a relatively poor fit for the data: *χ*^2^ (20, *N* = 253) = 97.12, *p* < 0.001, *χ*^2^/*df* = 4.85, CFI = 0.91, TLI = 0.88, SRMR = 0.06, and RMSEA = 0.12, 90% confidence interval (CI) [0.10, 0.20]. However, an examination of the modification indices indicated that conceptually and empirically meaningful correlated error terms should be correlated: (a) Item *e*1 with Item *e*2; (b) Item *e*2 with Item *e*6; and (c) Item *e*4 with Item *e*5. Our re-specified unidimensional model revealed an excellent model fit with the following indices: *χ*^2^ (17, *N* = 253) = 50.191, *p* < 0.001 is significant, *χ*^2^/*df* = 2.95 (smaller than 3), CFI of 0.96 (>0.95), SRMR of 0.04 is (<0.05), and RMSEA of 0.08 (90% CI [0.06, 0.11]) [<0.08], with significant factor loading ranging from 0.66 to 0.74, as seen in Figure 1.

### 3.4. Reliability

The internal consistency coefficient (Cronbach’s alpha) for the RACSS was computed to be 0.89, indicating good reliability in a sample of rural veterans.

### 3.5. Association Between RACSS and Minority Status, Willingness to Seek Psychological Services, and Number of Physical and Mental Health Conditions

An independent sample *t*-test was conducted to compare the rural access to healthcare and services of rural veterans from minoritized racial and ethnic groups and non-minority rural veterans. Significant differences were found in the access to healthcare and services for rural veterans between minoritized racial and ethnic groups (*M* = 3.51, *SD* = 0.79) and non-minority rural veterans (*M* = 3.22, *SD* = 0.83); *t*(498) = 3.48, *d* = 0.35 *p* < 0.001, indicating that rural veterans from minoritized racial and ethnic groups experience significantly higher levels of difficulties in accessing rural healthcare and services. Correlation analysis revealed that difficulties in accessing rural healthcare and services were positively associated with willingness to see a therapist for psychological help (*r* = 0.12, *p* < 0.05) and number of mental health conditions (*r* = 0.09, *p* < 0.05). Interestingly, difficulties in accessing rural healthcare and services were negatively associated with the number of physical health conditions (*r* = −0.10, *p* < 0.05). Please see Table 2 for detailed results.

## 4. Discussion

The present study aimed to develop and validate the RACSS among rural veterans, with particular attention to those with chronic health conditions. The results underscore the critical barriers to healthcare access faced by rural veterans [3,4,9], particularly minority groups. The findings provide valuable insights into the challenges of healthcare delivery in rural settings and highlight areas where targeted interventions are needed.

The validation of the RACSS demonstrated acceptable psychometric properties, including high internal consistency reliability (α = 0.89) and a well-fitting one-factor structure. These results are consistent with previous studies that have identified significant challenges in access to healthcare and services among rural populations [2], especially veterans who may have unique healthcare needs due to their service-related injuries and chronic health conditions [5].

Furthermore, our findings identified that rural veterans from minoritized racial and ethnic groups experience significantly higher levels of difficulty in accessing healthcare and services compared to their non-minority counterparts. This aligns with existing research that highlights racial and ethnic disparities in healthcare access [17], which are often exacerbated in rural settings due to limited resources and infrastructural challenges. Therefore, in addition to the general barriers to healthcare and services access experienced in rural areas, it is crucial to address the specific challenges faced by veterans from minoritized racial and ethnic groups within these interventions.

Finally, our correlation analysis revealed that difficulties in accessing rural healthcare and services were positively associated with both the willingness to see a therapist for psychological help and the number of mental health conditions. This suggests that individuals with a higher number of mental health conditions and a greater willingness to seek help reported more difficulties in accessing rural healthcare and services. Increased investment in rural mental health services is important to meet the growing mental health needs of rural veterans. This may involve expanding telehealth services, increasing the number of mental healthcare providers in rural locations, and reducing the stigma associated with mental illness.

Interestingly, difficulties in accessing rural healthcare and services were negatively associated with the number of physical health conditions, indicating that individuals with more physical health conditions reported fewer difficulties in accessing rural healthcare and services. This might seem counterintuitive, but individuals with more physical health conditions may already be engaged with the healthcare system, potentially receiving regular treatment or living in areas with better access to services. Therefore, it could be tested whether mental healthcare services can be integrated with physical health services as part of an integrated care strategy. This approach may help rural veterans identify and address mental health needs during routine physical health check-ups and facilitate timely referrals to mental health specialists.

The study’s limitations should also be acknowledged. The use of a survey distributed through media materials may have introduced selection bias, as participants who are more engaged with technology and media may be overrepresented in the sample. Additionally, while the RACSS demonstrated strong psychometric properties, further validation in diverse rural populations is needed to ensure its generalizability. One important limitation is the reliance on self-reported data. For example, the definition of “living in a rural area in the US” was not explicitly detailed, potentially leading to inconsistencies in interpretation among respondents. Next, this study did not have a comprehensive assessment of face and content validity for the instrument. While we conducted a literature review to identify barriers and challenges faced by rural veterans, we did not engage in extensive consultations with the target population or experts in the field. This may affect the relevance and comprehensiveness of the items included in the instrument. Finally, the decision to limit the number of items was influenced by the need for brevity, given the time constraints faced by respondents and the shortage of healthcare providers in rural locations. Additionally, the instrument lacks a strong theoretical foundation in its development process, which could enhance its robustness. Therefore, future research should aim to conduct more thorough validity assessments and consider established models.

Future research should explore the implementation of interventions designed to improve access to care in rural areas, particularly for veterans from minoritized racial and ethnic groups. These may include an expansion and systematic evaluation of community care programs [8,24]. These could also include telehealth services, mobile clinics, and transportation assistance, which have been shown to be effective in overcoming some of the barriers identified in this study [9,10,11]. Moreover, interventions may also focus on addressing language barriers, sociocultural factors (e.g., stigma), and the cultural competency of service providers, as these factors contribute to disparities in healthcare access and services for veterans from minoritized racial and ethnic groups [18]. Lastly, policy initiatives aimed at reducing healthcare disparities in rural areas should be informed by the findings of this study, particularly the significant challenges faced by veterans from minoritized racial and ethnic groups, and those with disabilities.

## 5. Conclusions

In conclusion, this study contributes to the growing body of literature on rural access to healthcare and services and provides a validated tool—the RACSS—for assessing access to care and services among rural veterans. The findings from this study highlight the urgent need for interventions and policies that address the rural healthcare needs of this underserved population, with particular attention to minority groups.

## Figures and Tables

**Table 1:** Factor loadings, communalities, and means of items and total score, and reliability information.

Item	Factor Loading	*h* ^2^	*M*
Due to living in a rural area, I have not been able to:			
Item 1	0.76	0.57	3.30
Item 2	0.74	0.54	3.35
Item 3	0.69	0.48	3.39
Item 4	0.76	0.57	3.20
Item 5	0.76	0.57	3.19
Item 6	0.74	0.54	3.32
Item 7	0.77	0.59	3.41
Item 8	0.78	0.61	3.31
Total *M* and *SD*	3.31 (0.83)		
Eigenvalue	4.48		
% Variance	55.93		
Cronbach’s alpha	0.90		

**Table 2:** Group Differences and Correlation Analysis Results.

**Group** **Differences**	**Group Variables**	**M (SD)**	***t***	**Effect Size**	
	Minority	3.51 (0.79)	3.48	*d* = 0.35	
	Non-minority	3.22 (0.83)			
**Correlation**	**Variables**	1	2	3	4
	1. Rural Access to Healthcare and Services	-	0.12 *	0.09 *	−0.10 *
	2. Willingness to Seek Psychological Help		-	0.19 *	−0.02 ^+^
	3. Number of Mental Health Conditions			-	0.23 *
	4. Number of Physical Health Conditions				-

* *p* < 0.05, ^+^ *p* = n.s.

## Data Availability

Data may be available upon request.

## Supplementary Materials

The following supporting information can be downloaded at: https://www.mdpi.com/article/10.3390/healthcare13030275/s1, File S1: The Rural Access to Care and Services Scale (RACSS).
