# Supplementary material for: Access to Care and Services Among U.S. Rural Veterans With and Without Disabilities: A National Study
Source: Healthcare (Basel). 2025 Jan 30;13(3):275. doi: 10.3390/healthcare13030275 (PMC11817750; doi:10.3390/healthcare13030275)
Supplement: Supplementary file 1 [file healthcare-13-00275-s001.zip › healthcare-3327159-supplementary.pdf]

The Rural Access to Care and Services Scale (RACSS)

Overview: Access to care and services is a critical issue for rural Veterans, particularly those managing chronic health conditions. While previous research has examined rural healthcare access broadly, specific barriers faced by rural Veterans have been less explored. The *Rural Access to Care and Services Scale* (RACSS) was developed to fill this gap, offering a reliable and valid tool to measure the perceived difficulties in accessing healthcare and services faced by rural Veterans.

Instruction for Use: The RACSS is designed to be administered to rural Veterans to assess their perceived barriers to accessing healthcare and services. The scale consists of several items related to key areas such as access to mental health care. Each item is rated on a 5-point Likert scale, where: 1 = Strongly Disagree to 5 Strongly Agree, with higher scores indicating lower levels of access to care and services. Respondents are asked to rate the extent to which they agree or disagree with statements about the difficulties they encounter in accessing services due to their rural location. Please note that some wordings changed from what was originally being used to increase clarity.

Items:

Due to living in a rural area, I have not been able to:

1. access physical health care services.
2. access mental health care services.
3. participate in outside activities I am interested in.
4. exercise or physically active.
5. access healthy food.
6. access my friends and family.
7. access transportation services.
8. access internet or phone services

Scoring and Interpretation: The total score is the sum of responses, with possible scores ranging from 8 to 40, where higher scores indicate greater perceived difficulties in accessing healthcare and services due to rural living. For example, a score of 8 reflects minimal difficulty in accessing services, while a score of 40 indicates significant challenges.

Psychometric Properties and Limitations: The RACSS was developed through a comprehensive process involving the collection of data from 500 rural veterans. The scale was rigorously tested using Exploratory Factor Analysis (EFA) and Confirmatory Factor Analysis (CFA) to establish its factor structure. The analysis revealed a one-factor structure, meaning all items on the scale measure a single underlying construct of access to healthcare and services. The scale demonstrates excellent internal consistency (Cronbach's alpha = 0.89) and a one-factor structure, making it a reliable tool for assessing rural access barriers. It is particularly useful in research and policy

development, as it helps identify areas where rural veterans face the greatest challenges and informs targeted interventions aimed at improving healthcare access. This tool can also help policymakers and healthcare providers develop strategies to reduce disparities and enhance the quality of life for rural veterans and other underserved populations. However, while the RACSS demonstrated strong psychometric properties, further validation in diverse rural populations is needed to ensure its generalizability. The study also did not conduct a comprehensive assessment of face and content validity for the instrument. Although a literature review was conducted to identify barriers faced by rural veterans, there was no extensive consultation with the target population or subject matter experts, which may impact the relevance and comprehensiveness of the items included. Furthermore, the decision to limit the number of items was influenced by the need for brevity, given the time constraints faced by respondents and the shortage of healthcare providers in rural areas. Lastly, the instrument lacks a strong theoretical foundation, which could enhance its robustness. Therefore, future research should aim to conduct more thorough validity assessments and consider established models to further refine and validate the scale.
